# Marijuana's potential in neurodegenerative diseases: an editorial

**DOI:** 10.3934/Neuroscience.2023014

**Published:** 2023-06-25

**Authors:** Arosh S. Perera Molligoda Arachchige

**Affiliations:** Department of Biomedical Sciences, Humanitas University, Milan, Italy

Neurodegenerative diseases, such as Alzheimer's disease, Parkinson's disease, Huntington's disease, and amyotrophic lateral sclerosis (ALS), represent a significant global health challenge. These conditions are characterized by the progressive deterioration of neurons in the central nervous system, leading to debilitating symptoms and a decline in cognitive and motor functions. Despite extensive research, effective treatments that can halt or reverse disease progression remain elusive. In recent years, there has been increasing interest in exploring the potential role of marijuana in the management and treatment of neurodegenerative diseases [1]. Inspired by the Special Issue, “Special Edition on Marijuana” of *AIMS Neuroscience*, we would like to provide a thoughtful perspective on marijuana's potential and its implications in the field of neurodegenerative research.

Marijuana is a plant that contains numerous chemical compounds, with two primary constituents gaining significant attention: tetrahydrocannabinol (THC) and cannabidiol (CBD). THC is known for its psychoactive properties such as hypolocomotion, hypothermia, catalepsy, and analgesia. On the other hand, THC has neuroprotective, antispasmodic, and anti-inflammatory actions, which are mediated through the activation of different receptors, such as CB2 and PPARγ [2],[9]. Until recently, most of the interest in medical uses of marijuana was focused on the actions of THC, but now there is growing interest in potential medical uses of CBD, which does not have the psychoactive effects of THC and appears to have a number of therapeutic benefits. Other cannabinoids and terpenes present in marijuana contribute to its diverse pharmacological profile, potentially influencing its effectiveness in neurodegenerative diseases. Understanding the composition and interactions of these compounds is crucial in assessing marijuana's therapeutic potential [2].

Interestingly, emerging evidence suggests that marijuana and its constituents possess neuroprotective properties. Cannabinoids, through their interaction with the endocannabinoid system, have demonstrated anti-inflammatory, antioxidant, and anti-excitotoxic effects, which may help combat the underlying mechanisms of neurodegeneration. Preclinical studies have shown promising results, indicating that marijuana-based compounds can protect neurons from damage, promote neuronal survival, and enhance neuroplasticity. These findings warrant further investigation to elucidate the precise mechanisms and translate them into effective clinical interventions [3].

Furthermore, clinical studies evaluating marijuana's effects on neurodegenerative diseases are limited but have shown promising results. In Alzheimer's disease, cannabinoids have exhibited potential in reducing neuroinflammation, improving cognitive function, and modulating neurogenesis [4]. In Parkinson's disease, marijuana-based treatments have demonstrated benefits in alleviating motor symptoms, improving sleep quality, and enhancing overall quality of life [5]. Preliminary studies in Huntington's disease and ALS have also indicated potential therapeutic effects, although more research is needed [6]. These findings highlight the need for robust clinical trials to establish marijuana's efficacy, optimal dosage, and long-term safety profile.

While marijuana shows promise, several challenges need to be addressed. The legal and regulatory landscape surrounding marijuana poses barriers to conducting clinical trials and implementing standardized treatment approaches [7]. Additionally, the potential side effects, drug interactions, and variations in individual responses to marijuana-based treatments require careful consideration [8]. Furthermore, the development of targeted therapies based on specific neurodegenerative disease subtypes and stages demands a deeper understanding of the complex pathophysiology of each condition.

Moving forward, collaborative efforts involving researchers, clinicians, policymakers, and patients are essential to advance our understanding of marijuana's role in neurodegenerative diseases. Large-scale clinical trials with rigorous methodologies are needed to evaluate the safety, efficacy, and long-term effects of marijuana-based treatments. Standardization of formulations, dosages, and delivery methods is crucial to ensure consistent and reliable outcomes. Additionally, innovative approaches such as precision medicine and personalized treatment strategies may help optimize the implementation of marijuana-based therapies based on individual patient characteristics and disease progression.

The potential role of marijuana in neurodegenerative diseases is a topic that warrants careful consideration and further investigation. And, we believe this special issue of *AIMS Neuroscience* is one the perfect venues to spark evidence-based discussion on this topic. While the current scientific evidence is promising, more research, including well-designed clinical trials, is needed to establish marijuana's efficacy, safety, and optimal usage in the treatment of neurodegenerative conditions. Collaborative efforts between researchers, healthcare professionals, regulatory bodies, and patients are crucial in advancing our understanding and exploring the full potential of marijuana-based therapies. By embracing rigorous scientific exploration, we can unlock new possibilities and potentially improve the quality of life for individuals living with neurodegenerative diseases. However, it is essential to balance the excitement surrounding marijuana's potential with responsible and evidence-based approaches to ensure safe and effective treatments in this complex field.
